# The serotonin transporter and the serotonin 1B receptor in relation to social cognition in healthy adults

**DOI:** 10.1186/s13550-026-01435-7

**Published:** 2026-04-17

**Authors:** Gina Griffioen, Ämma Tangen, Emma Veldman, Jonas E Svensson, Mikael Tiger, Magdalena Nord, Max Andersson, Kimmo Sorjonen, Andrea Varrone, Christer Halldin, Katarina Varnäs, Jacqueline Borg, Johan Lundberg

**Affiliations:** 1 Centre for Psychiatry Research and Centre for Cognitive and Computational Neuropsychiatry (CCNP), Department of Clinical Neuroscience, Karolinska Institutet & Stockholm health care services, Region Stockholm, Stockholm, 113 64 Sweden; 2 Centre for Psychiatry Research, Department of Clinical Neuroscience, Karolinska Institutet & Stockholm health care services, Region Stockholm, K8 CPF Farde/Halldin, 171 77, Stockholm, Sweden; 3 Department of Clinical Neuroscience, Division of Psychology, Karolinska Institutet, K8 Psykologi Melin, 171 77, Stockholm, Sweden

**Keywords:** Social cognition, Positron Emission Tomography, Serotonin transporter (5-HTT), Serotonin 1B receptor (5-HT_1B_), Transdiagnostic, Healthy subjects

## Abstract

**Background:**

Social cognition is impaired across multiple psychiatric disorders and varies dimensionally in the general population. Studying healthy adults can therefore inform mechanisms relevant for social cognition. This study aimed to extend prior findings of associations between social cognition and serotonin transporter (5-HTT) within autistic and non-autistic controls to healthy adults, and to examine serotonin 1B (5-HT_1B_) receptor binding.

**Results:**

Thirty-one healthy adults (15 males, 16 females) underwent PET imaging with [¹¹C]MADAM to quantify 5-HTT binding. In the replication cohort (*n* = 17), MASC performance correlated positively with putaminal 5-HTT binding (ρ = 0.61, *p* = 0.011, BFR = 15.2) and negatively with brainstem binding (ρ = −0.64, *p* = 0.008, BFR = 6.1). A similar positive association with putaminal 5-HTT binding was observed in the pooled sample (*n* = 31, ρ = 0.53, *p* = 0.003). However, no correlations survived correction for multiple comparisons. In a separate sample of 32 healthy adults (13 males, 20 females) examined with [¹¹C]AZ10419369 to assess 5-HT_1B_ receptor binding, no significant associations with measures of social cognition or central coherence were found.

**Conclusions:**

Results conceptually replicate an association between putaminal 5-HTT binding and social cognition in healthy adults, supporting a role of 5-HTT—but not 5-HT_1B_—in social cognitive processes.

## Background

As human beings, social cognition has played a pivotal role in the evolution of our species [1, 2]. Social cognition refers to a complex set of mental abilities underlying social stimulus perception, processing, interpretation, and response [3], and is an important determinant of mental health [4, 5] and emotional problems [6, 7]. Given the involvement of social cognition across multiple psychiatric conditions [8–16] and its normal distribution in the general population [17, 18], employing a dimensional approach promises a deeper understanding of social cognition’s role in psychiatric conditions. Being one of the constructs within The Research Domain Criteria (R-DoC) framework [19], studying social cognition might facilitate the identification of biomarkers associated with this trait and thereby enhancing the development of more precise diagnostic tools and treatments.

A central aspect of social cognition is Theory of Mind (ToM): the cognitive capacity to attribute mental states—such as beliefs, intentions, desires, emotions, and knowledge—to oneself and others, and to understand that these mental states may differ between individuals. While deficits in ToM long has been established as core feature in individuals with autism and their relatives [20, 21], poorer performance in ToM tests has also been observed in persons with schizophrenia and their siblings [9, 11], in those with bipolar disorder and their first-degree relatives [22, 23], and in subjects with depression - even during remission [24, 25]. Consequently, variations in social cognition are evident not only within specific psychiatric conditions, but also within healthy relatives and the general population, underscoring the relevance to study biomarkers for social cognition within a sample of healthy subjects.

In a study published by our group, we employed the molecular imaging technique positron emission tomography (PET) with the radioligand [^11^C]MADAM to quantify serotonin transporter (5-HTT) binding in vivo. This investigation revealed significantly lower 5-HTT binding in 15 participants with autism diagnosis compared to 15 controls, with reductions of 14.6% in total gray matter and similar decreases observed across cortical regions, subcortical areas, and in the brain stem [12]. Beyond these group differences, we identified meaningful correlations between 5-HTT binding and social cognition performance across the combined sample of individuals with and without autism. Specifically, 5-HTT availability correlated positively to performance on the Reading the Mind in the Eyes Test (RMET) in total gray matter, brain stem and 7 of 18 subregions, including nucleus accumbens. In addition, performance on Movie for Assessment of Social Cognition (MASC) and Faux Pas also correlated to 5-HTT availability in nucleus accumbens and putamen, whereas Faux Pas performance also correlated positively with anterior cingulate cortex 5-HTT binding. However, only the correlation between RMET and 5-HTT availability in anterior cingulate cortex survived correction for multiple comparisons. Importantly, these correlation patterns were also observed when analysing the non-autistic control participants independently.

These findings align with another PET study that reported positive correlations between Faux Pas performance and 5-HTT availability within several parts of the cingulate cortex in autistic participants [26], suggesting consistent involvement of the serotonin system in social cognitive processes across different populations.

In addition, previous research has demonstrated that healthy participants demonstrated enhanced facial expression recognition and faster response times following acute administration of selective serotonin reuptake inhibitors (SSRIs, which block 5-HTT) [27], while tryptophan depletion—which reduces serotonin levels—led participants to perceive social relationships as less intimate [28]. Moreover, involvement of the central serotonin system has been implicated in several of the above-mentioned psychiatric disorders, including autism [12, 29], schizophrenia [30], depression [31–33], ADHD [34], but also in borderline personality disorder [35], anorexia nervosa [36], and OCD [37]. Altogether, these findings indicate that serotonin is relevant for social cognition, even beyond the traditional diagnostic boundaries.

Similar to the observed correlations between 5-HTT availability and social cognition in our mixed sample of autistic and non-autistic participants, we identified correlations with central coherence - both an autistic trait and a transdiagnostic endophenotype characterized by detail-focused processing, rather than the whole picture (weak central coherence). In our previous study, we observed two correlations [12], which did not survive correction for multiple comparisons: performance on the Embedded Figures Test correlated with 5-HTT availability in nucleus accumbens and insula, while no correlations were found for the Fragmented Pictures Test.

Weak central coherence, like social cognition impairments, are well-documented in individuals with autism and their relatives [38, 39]. However, altered central coherence extends beyond the autism spectrum, manifesting in obsessive compulsive disorder (OCD) [40], in individuals with borderline traits [14], and eating disorders, including among their unaffected family members [41]. Importantly, central coherence varies continuously within the general population [17, 42], supporting its conceptualization as a dimensional trait, rather than a categorical deficit within the autistic population. Given our exploratory finding suggesting a possible relationship between central coherence and 5-HTT in a mixed clinical sample, confirmation of this association within a sample of solely healthy subjects represents a critical next step in establishing the generalizability of this neurobiological mechanism within the general population.

*Aims*.

Building upon our previous explorative finding, where higher 5-HTT binding correlated with enhanced performance on tests for social cognition and central coherence across both autistic and healthy adults [12], the first aim was to replicate this within a sample of solely healthy volunteers. Given that both social cognition and central coherence exhibit a normal distribution in the general population [17], we hypothesize that higher 5-HTT binding would predict superior performance in tests for both social cognition and central coherence.

To further investigate the serotonergic bases of these endophenotypes, we extended our research to examine a specific serotonin receptor subtype. 5-HT exerts its diverse effects through 14 receptor subtypes [43], with extracellular 5-HT being transported back into neurons by the 5-HTT. 5-HTT serves as a global marker of serotonergic function, and selective serotonin reuptake inhibitors (SSRI), targeting 5-HTT, do not significantly affect social cognition or central coherence [44], suggesting that specific receptor subtypes may be more directly involved in these processes. Given the limited availability of radioligands for human neuroimaging of the serotonin system, we focussed our investigation on the 5-HT_1B_ receptor. This receptor subtype represents a particularly relevant target for several reasons. First, both 5-HTT and 5-HT_1B_ serve as regulators of serotonin concentrations in the synapse [45], positioning them as key candidates for testing whether the serotonin hypothesis of autism can be extended to social cognitive processes. Second, 5-HT_1B_ receptors are highly expressed in cortical regions implicated in social cognition and central coherence, making them mechanistically plausible mediators for these functions. To our knowledge, this represents the first study investigating a potential relationship between 5-HT_1B_ and social cognition or central coherence in humans. Based on previous findings demonstrating correlations between 5- HT_1B_ and 5-HTT availability within the cortical regions [46], we hypothesized that higher 5-HT_1B_ binding would correlate to superior performance on measures of social cognition and central coherence, particularly in cortical regions.

## Methods

### Participants

All participants were enrolled as control subjects in four independent PET studies between 2013 and 2017 [12, 46–48]. A total of 47 control subjects were pooled, of which 17 participants were investigated with both radioligands. For analysis of 5-HTT binding, 31 participants were included, comprising 15 control participants from study [12] and 17 from study [46]. For analysis of 5-HT_1B_ receptor binding, 33 participants were included, comprising the same 17 controls from study [46], 4 controls from study [47] and 12 controls from study [48]. Given the resource-intensive nature of PET studies, it is standard practice to compile such a database and administer a consistent test battery. All studies were approved by the Regional Ethical Review Board in Stockholm and by the Radiation safety committee of the Karolinska University Hospital. All participants provided written informed consent.

### Inclusion criteria

All participants underwent a comprehensive assessment to ensure their eligibility. This included a clinical interview using the Mini International Neuropsychiatric Interview (M.I.N.I.), a physical examination conducted by a medical doctor, and negative results in urine toxicology screenings before the PET examinations. Exclusion criteria encompassed a medical history of substance abuse, chronic psychiatric disorders, previous head trauma, brain pathology detected via magnetic resonance imaging (MRI), significant medical conditions, and pregnancy. The recruitment and examination of participants were conducted by members of the PET research group at Karolinska Institutet, Stockholm, Sweden.

### Assessment of social cognition and central coherence

The study assessed the dimensional aspect of social cognition through various social-cognitive tests. Behavioural testing was scheduled to coincide with the PET examination on the same day; however, in a small number of participants from diffferent studies, assessments were conducted a few days after PET due to logistical constraints. These assessments included the Movie for Assessment of Social Cognition (MASC [49]), Reading the Mind in the Eyes Test (RMET [50]) and Faux Pas [51]. The MASC test involves a 15 min video portraying four characters at a dinner party. It aims to assess ToM by posing questions related to the thoughts, beliefs and intentions of the characters. The RMET task requires participants to discern the appropriate emotion expressed by each eye pair from four alternatives. Faux Pas assesses ToM through inquiries about social situations and unintentional social rule violations. For the assessment of central coherence, two visual tasks, one focusing on global perception and the other on local details, were employed. The Fragmented Pictures Test (PFT [52], , required participants to identify progressively revealed drawings of common objects with as little information as possible. The Embedded Figures Test (EFT [53], , tasks participants with detecting simpler shapes embedded within larger and more complex pictures.

### Image acquisition and analysis

Magnetic resonance imaging (MRI; 3T, GE Healthcare) was conducted for exclusion of brain anomalies and for delineation of brain regions. For each participant, the MR image was co-registered to a summated PET image as previously described using Statistical parametric mapping (SPM12; Wellcome Department of Cognitive Neurology, University College, London, U.K).

During PET examinations, subjects were positioned recumbent with their heads inside the PET system and wore a plastic helmet to minimize head movement. Radioligands [^11^C]MADAM and [^11^C]AZ10419369 were synthesized as previously reported [54, 55].

#### 5-HTT sample

14 control subjects from the original study underwent examination of 5-HTT binding using [^11^C]MADAM and the High Resolution (HR) ECAT PET system (Siemens, Knoxville, TN), while the additional 17 subjects were examined with the High Resolution Research Tomograph (HRRT) ECAT PET system (Siemens Molecular Imaging). The acquisition time for all examinations was 93 min. The PET data from the 14 subjects investigated with the HR system were divided into 31 time frames (4 × 15 s, 4 × 30 s, 6 × 60 s, 6 × 180 s, and 11 × 360 s), reconstructed using filtered back projection, and corrected for head motion using a frame-to-first-frame approach. For the 17 subjects investigated with the HRRT PET system, the PET data was divided into 38 frames (9 × 10 s, 2 × 15 s, 3 × 20 s, 4 × 30 s, 4 × 60 s, 4 × 180 s, and 12 × 360 s). Dynamic PET images were corrected for head motion using a between frame-correction algorithm implemented in SPM12 (Wellcome Department of Cognitive Neurology, University College, London, UK). Injected radioactivity and molar (specific) activity were within previously reported ranges for these datasets. Injected activity was typically ~ 370–410 MBq, with molar activity in the range of approximately 230–240 GBq/µmol at the time of injection [12, 46].

For delineation of brain regions in the 5-HTT sample, Freesurfer (versions 5.0 and 6.0 [56]) was employed, and brain region anatomy was defined using the cortical atlas of Desikan-Killany [57]. 5-HTT binding potential (BP_ND_) was quantified using the simplified reference tissue model (SRTM [58]), with the cerebellar gray matter serving as the reference region [59, 60]. For the replication sample, this differs from the quantification methods previously reported [46]. Regions of interest (ROIs) were selected based on where correlations between social cognition and 5-HTT were found in our original study, and included gray matter, putamen, brain stem, frontal cortex, anterior cingulate cortex, posterior cingulate cortex, insula, amygdala, and nucleus accumbens.

#### 5-HT_1B_ receptor sample

33 subjects underwent examination of 5-HT_1B_ receptor binding using the radioligand [^11^C]AZ10419369 and the HRRT PET system. Data from the first 63 min were used for quantification to enable data pooling, to ensure consistent frame definitions across all data. Injected radioactivity and molar (specific) activity were consistent with previously published values. Injected activity was typically ~ 380–414 MBq, with molar activity in the range of approximately 265–330 GBq/µmol at the time of injection [46–48]. Brain regions were automatically defined using Freesurfer (version 6.0 [56]), except for the dorsal brainstem (DBS), which was defined based on [^11^C]AZ10419369 PET template data [61, 62], and gray matter defined by SPM segmentation. The cerebellum, with negligible 5-HT_1B_ receptor density [63], served as the reference region for both BP_ND_ quantification using SRTM and an algorithm for wavelet-aided parametric imaging (WAPI) to reduce noise [64] for smaller ROIs, including the amygdala, and hippocampus. ROIs were selected based on existing literature related to social cognition and 5-HTT, and included gray matter, putamen, brain stem, striatum, frontal cortex, temporal cortex, anterior cingulate cortex, posterior cingulate cortex, insula, orbitofrontal cortex, caudatus, thalamus, nucleus accumbens, hippocampus, occipital cortex, amygdala, and pallidum.

### Statistical analysis

Descriptive statistics for both samples are presented in Table 1.

**Table 1:** Descriptive statistics for the samples examined with [^11^C]MADAM (5-HTT sample) and [^11^C]AZ10419369 (5-HT_1B_ sample)

	5-HTT sample			5-HT_1B_ receptor sample		
	*n*	Range	Mean (SD)	*n*	Range	Mean (SD)
Age	31	21–75	40.7 (13.8)	33	20–75	38.3 (14.91)
Sex assigned at birth						
Female	16			20		
Male	15			13		
MASC	31	27–42	35.2 (4.1)	33	25–42	34 (4.34)
RMET	31	20–33	28.2 (3.3)	32	20–32	28 (3.24)
Faux Pas	31	41–60	54 (4.9)	17	46–60	55 (4.55)
EFT	31	31-1509	473 (322)	33	31-1509	514 (330.44)
FPT	31	80–460	185 (79.8)	33	87–538	197 (87.59)

5-HTT serotonin transporter, 5-HT_1B_ serotonin 1B receptor, MASC movie for assessment of social cognition, RMET reading mind in the eyes test, EFT embedded figure test (seconds), FPT fragmented picture test (seconds), *n* number of participants, *SD* standard deviation

Due to the non-normally distributed test data, Spearman’s correlations were employed to explore inter-regional relationships, associations between behavioural tasks and demographic variables (age and education), as well as associations between radioligand binding and demographic variables within each sample, corrected for age. Given the partially exploratory nature of the study, and for ease of interpretation, findings are presented without correction for multiple comparisons. In addition, the false discovery rate [65] was applied. All statistical tests were two-tailed, and the significance level was set at *p* ≤ 0.05 and were corrected for multiple comparisons using Benjamini-Hochberg method [65]. Statistical analyses were conducted using R Studio [66]. Raw data and code are available at https://osf.io/z6mbn/?view_only=d4028579be2143d3b077f408f65e78ce.

#### 5-HTT sample

Given the inclusion of the 14 controls from our original study [12], where correlations between social cognition or central coherence and 5-HTT binding were identified, and the potential confounding factors related to disparities in PET systems, several methodological steps were taken.

First, an analysis by excluding the 14 subjects from the original study was conducted, resulting in a distinct replication sample comprising 17 new subjects examined using the same PET system. Spearman’s correlations were employed for this replication sample, as well as for the healthy controls from of the original study (where autistic subjects also were included in the analysis).

Subsequently, to provide a robust assessment of the correlation’s replicability, we employed Bayesian analysis, which yielded a Bayes Replication Factor. This test compares the predictive adequacy of the null hypothesis (H_0_ (i.e., no correlation) versus the alternative hypothesis (H_r_, representing the original correlation). The Bayes Replication Factor quantifies the likelihood of observing the original correlation versus no correlation, given the additional data [67, 68]. Importantly, this approach allows for the integration of additional data, providing a comprehensive evaluation of the correlation’s replicability.

Lastly, BP_ND_ values were standardized (with mean = 0 and standard deviation = 1) within both groups to address potential variations arising from the utilization of the different PET systems. Then, the Spearman’s correlation test was conducted for the whole sample.

These methodological steps were taken to ensure the reliability and validity of our findings, considering the potential impact of varying PET systems and the specific composition of the study population.

#### 5-HT_1B_ receptor sample

Partial Spearman´s correlations, corrected for age, were used to investigate associations between cognitive performance and 5-HT_1B_ BP_ND_ values. For non-significant correlations, a Bayes Factor for correlation was calculated, to quantify evidence in favor of the null hypothesis (i.e. no correlation) [68, 69] .

During the preparation of this work, the author(s) used ChatGPT-3.5 (OpenAI, 2024) to improve coding guidance, readability, and language, as well as Claude.ai (Anthropic, 2024) to improve readability and language. After using this tool/service, the author(s) reviewed and edited the content as needed and take(s) full responsibility for the content of the published article.

## Results

### 5-HTT sample

#### Correlations between 5-HTT binding and performance on social cognition tests

##### Controls from the original sample

In the control participants from the original sample (*n* = 14), significant positive correlations were observed between RMET test scores and 5-HTT binding across several limbic and cortical regions: anterior cingulate cortex (Spearman’s rho = 0.72, *p* = 0.012), insula (Spearman’s rho = 0.64, *p* = 0.033), and posterior cingulate cortex (Spearman’s rho = 0.73, *p* = 0.010). However, none of these correlations remained statistically significant following correction for multiple comparisons, using the Benjamini-Hochberg procedure.

##### Replication sample

**Fig. 1:**
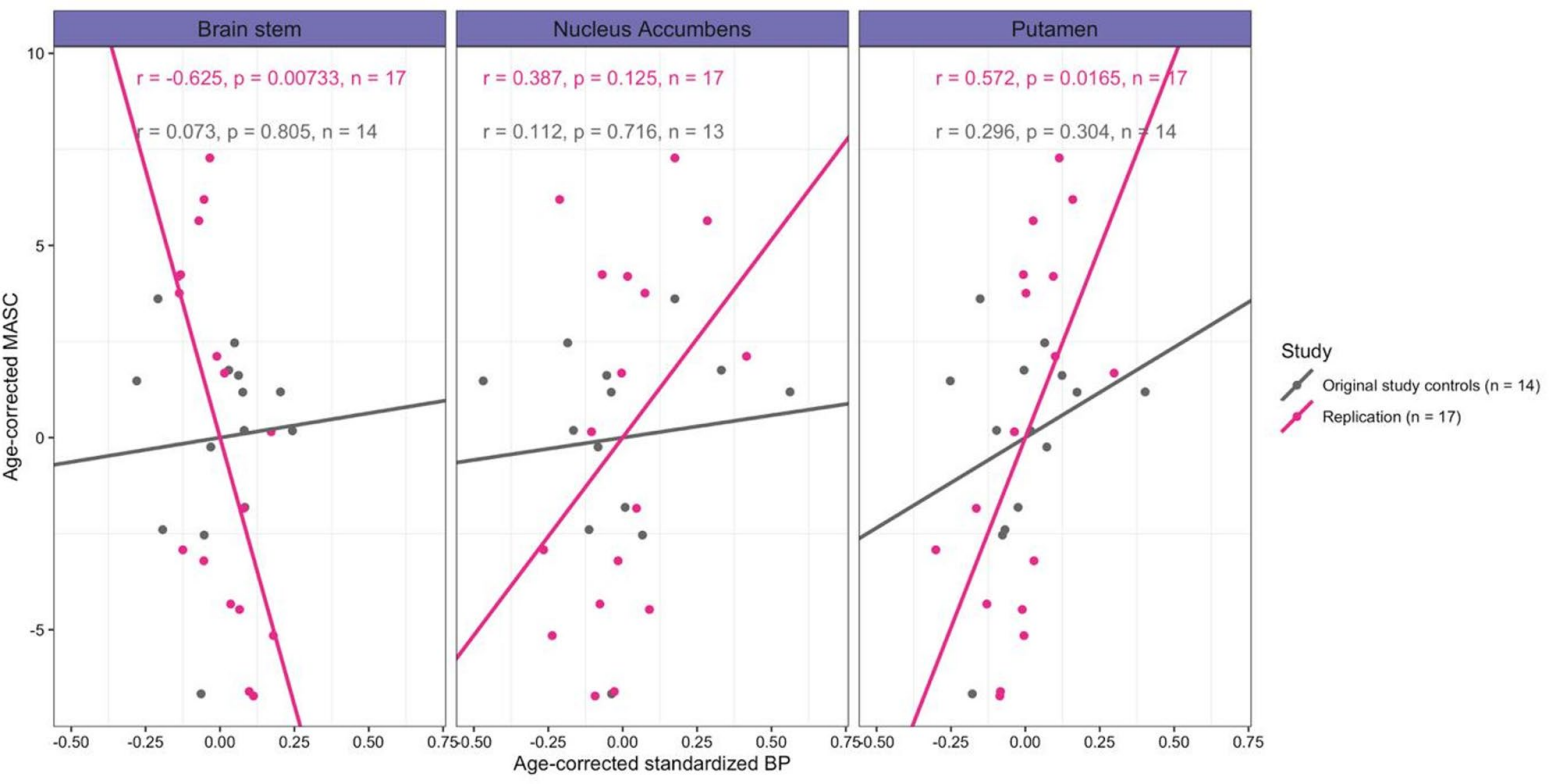
correlations between 5-HTT binding and MASC scores in control participants from original and replication sample. Partial pearsons correlations between 5-HTT binding potential (BP_ND_) and MASC scores in the control participants from the original sample and the replication sample, corrected for age. Abbrevations: MASC Movie for Assessment of Social Cognition, BP_ND_ binding potential

In the replication sample consisting of 17 participants, significant correlations were identified between MASC performance and 5-HTT availability in two brain regions. Specifically, positive correlations were observed in the putamen (Spearman’s rho = 0.61, *p* = 0.011), while a negative correlation was found in the brain stem (Spearman’s rho = -0.64, *p* = 0.008, Fig. 1). Similar to the results from the original sample, these correlations did not remain significant after correction for multiple comparisons, using the Benjamini-Hochberg procedure. No significant correlations were detected between 5-HTT binding on other social cognitive tests in the replication sample.

Figure 1 displays the correlations between 5-HTT BP_ND_ and MASC scores for both the original and replication sample, with separate panels illustrating findings in the brain stem, nucleus accumbens and putamen – regions where significant uncorrected correlations were identified in at least one sample. Note that the correlation in nucleus accumbens was previously observed in the original study when analysing the complete sample including participants with autism.

##### Bayes replication analysis

Replication Bayes Factors were computed for the correlations between test scores and 5-HTT BP_ND_ for regions where significant effects were observed in either study. For MASC performance, the probability that the data represent a replication of the correlation obtained in the original study is 15.2 times more likely than that of a correlation of 0 (BF_0r_ = 15.2) in the putamen, and 6.1 times more likely in the brain stem.

For the significant correlations observed in the original study (between performance on RMET and 5-HTT binding in anterior cingulate cortex, posterior cingulate cortex and insula, the Bayes Factors ranged from 0.02 to 0.16, indicating moderate to strong evidence in favour of the null-hypothesis (i.e. no correlation).

##### Pooled sample analysis

**Fig. 2:**
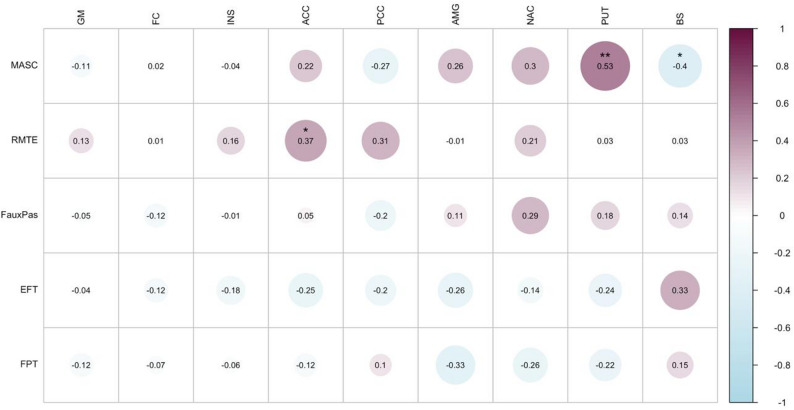
Correlations between 5-HTT binding and social cognitions and central coherence tests in the pooled sample. Spearman’s correlation between the standardized 5-HTT binding potential (BP_ND_) and performance on tests for social cognition and central coherence in the pooled sample of 14 controls from original study, plus 17 additional healthy subjects. Abbreviations: GM gray matter, PUT putamen, BS brain stem, FC frontal cortex, ACC anterior cingulate cortex, INS insula, AMG amygdala, PCC posterior cingulate cortex, NAC nucleus accumbens, MASC Movie for Assessment of Social Cognition, RMET Reading the Mind in the Eyes Test, EFT Embedded Figures Test, FPT Fragmented Pictures Test. * significant result (*p* < 0.05), before correction for multiple comparisons. None of the correlations remained significant after multiple comparison correction

Figure 2 illustrates the correlations between 5-HTT binding and performance on social cognition tasks for the pooled sample, containing control participants from the original study (*n* = 14) and the replication sample (*n* = 17). A positive correlation between 5-HTT BP_ND_ and MASC was observed in the putamen (Spearman’s rho = 0.53, *p* = 0.003), while a negative correlation was observed in brain stem (Spearman’s rho = -0.40, *p* = 0.028); For RMET and 5-HTT BP_ND_ in anterior cingulate cortex a positive correlation was observed (Spearman’s rho = 0.37, *p* = 0.04). None of these correlations remained significant after correction for multiple comparisons.

### Correlations between 5-HTT binding and performance on central coherence tests

No significant correlations between 5-HTT binding and performance on EFT or FPT were observed in the controls from the original sample (*n* = 14), the replication sample (*n* = 17), or in the pooled sample (Fig. 2). The original study, including autistic participants, found significant correlations between performance on EFT and 5-HTT binding in insula and nucleus accumbens. The Bayes Replication Factors were 0.17 and 0.19 for these regions, respectively, indicating that the absence of a correlation was (1/0.17 =) 5.9 and (1/0.19 =) 5.3 times more likely than the correlations observed in the controls from the original study.

### 5-HT_1B_ sample

#### Correlations between 5-HT_1B_ receptor binding and performance on tests for social cognition and central coherence

5-HT_1B_ receptor binding in thalamus correlated positively to EFT performance (Spearman´s rho = 0.41; *p* = 0.02) and negatively to performance on RMET (Spearmans rho = -0.45, *p* = 0.02). However, these correlations were no longer statistically significant after multiple comparison correction.

Bayesian analysis revealed mostly evidence in favor of the null hypothesis (i.e. no correlation). The Bayes Factors for correlation ranged from 0.015 to 4.5, with the majority falling below 1/3 indicating moderate evidence in favor of the null-hypothesis [70]. Only the correlations between thalamic 5-HTT binding and both EFT and RMET performance provided anecdotal and moderate evidence in favor of the alternative hypothesis (i.e. the presence of a correlation).

## Discussion

In the present study, we examined the relationship between social cognition and serotonergic markers in healthy adults using PET imaging of the 5-HTT and 5-HT_1B_ receptor. Building on our previous work in a mixed sample of autistic and neurotypical participants [12], we sought to evaluate the robustness and generalizability of previously reported associations within a non-clinical population. We replicated the positive correlation between performance on MASC and 5-HTT binding in the putamen in a sample of healthy adults, extending findings from our prior study that included both autistic and healthy participants [12]. Despite the correlation not retaining significance after correction for multiple comparisons, the Bayes Replication Factor of 15.2 indicates that the correlation found in the original study is 15.2 times more likely than the null hypothesis (no correlation), providing evidence for a genuine association [70].

However, our replication efforts were not uniformly successful. We were unable to replicate correlations between 5-HTT binding and social cognition in other brain regions, nor for the other tests for social cognition or central coherence. Notably, despite stronger and more numerous correlations for the RMET test in our original study, we could not replicate these findings in the current sample of healthy controls. This selective replication pattern may reflect differences in test sensitivity and score distributions within non-clinical populations, but alternative explanations, including limited statistical power, cannot be excluded.

In our original study, correlations between RMET performance and regional 5-HTT binding were primarily driven by autistic participants, whereas the MASC-5-HTT correlation was evident across both autistic and neurotypical participants. Examination of score distributions reveals that MASC performance shows greater variability among healthy controls compared to RMET performance (MASC: range 27–42, median 36; RMET: range 20–33, median 29). This superior score distribution for MASC may indicate that it is more sensitive to individual differences in social cognitive abilities within the general population, whereas RMET may show ceiling effects or restricted range in neurotypical samples. These findings suggest that different social cognition measures may have varying utility for detecting neurobiological correlates in clinical versus non-clinical populations. While RMET may be particularly sensitive to the more pronounced social cognitive differences observed in autism spectrum conditions, MASC appears better suited for capturing the subtler variations in social cognition present within neurotypical populations.

Furthermore, though we observed a correlation between tests for central coherence and 5-HTT binding in our previous study, we were unable to replicate this within the current sample of healthy controls. In contrast, we found evidence in favour of the null hypothesis (i.e. no correlation was about 5 times more likely than the correlation of the original study). These findings imply that 5-HTT is not involved in central coherence in healthy adults.

In contrast to our original findings, we observed a negative correlation for MASC scores and 5-HTT binding in brain stem, whereas the original study showed a positive correlation in this region. The Bayes Replication Factor of 6.1 indicates that the positive correlation observed in the original study is 6.1 times more likely than the null hypothesis of no correlation. Bayes Factors below 3 are typically considered to provide ambiguous support, whereas Bayes Factors above 10 are considered to offer strong support in favor of a hypothesis [70]. Notably, we failed to replicate the findings from Nakamura et al. [26] regarding the correlation between performance on Faux Pas within autistic participants and 5-HTT availability in several parts of the cingulate cortex, within a sample of healthy subjects. Altogether, these findings provide conceptual replication evidence for a possible role of putaminal 5-HTT in social cognition, within the general population, while the role of other brain regions remains less certain and warrants further investigation.

Although the literature on this topic is limited, several studies suggest a potential involvement of the putamen in social cognition. Lesion, neurodegenerative, and functional imaging studies have linked putaminal involvement to TOM performance, primarily through dopaminergic mechanisms [71–74]. While these findings primarily implicate dopaminergic mechanisms, serotonin may also play a crucial role in putaminal modulation of social cognition. The putamen represents one of three brain regions with the highest 5-HTT binding potential values, which may have provided sufficient statistical power to detect serotonergically-mediated correlations that were obscured in cortical regions where [^11^C]MADAM binding is substantially lower. However, this power-based explanation is challenged by by the absence of comparable findings in other high-binding regions, including the thalamus and pallidum. The putamen’s role in social cognition may involve complex interactions between dopaminergic and serotonergic systems within cortico-striatal circuits. Given the well-established co-regulation and functional interactions between these neurotransmitter systems [75], serotonergic modulation through 5-HTT could complement or modulate dopaminergic influences on social cognitive performance. In conclusion, while dopaminergic involvement in putaminal social cognition is better established, our findings suggest that serotonergic mechanisms may represent an important but underexplored pathway warranting further investigation.

In our exploratory part, we found that 5-HT_1B_ receptor binding in the thalamus showed opposing relationships with two measures: it correlated positively with performance on EFT, reflecting greater accuracy and detail-focussed processing, but negatively with RMET performance, which indexes the ability to infer mental states from eye-region photographs (i.e. superior social cognition). However, none of the observed correlations remained statistically significant after correcting for multiple comparisons. Bayesian analysis further indicated that the data provided mostly moderate evidence in favour of no correlation. The only exceptions were thalamic 5-HTT_1B_ binding with EFT and RMET performance, where the presence of a correlation was 2.8 and 4.5 times more likely, respectively, then the null hypothesis. While these results suggest a potential role for thalamic 5-HT_1B_ in both social cognition and central coherence, replication in larger, independent samples is required.

5-HTT and 5-HT_1B_ receptors are both expressed in serotonergic neurons, regulating presynaptic 5-HT release. However, 5-HT_1B_ receptors are also expressed in non-serotonergic neurons as heteroreceptors, regulating neurotransmitter release [45]. While 5-HTT binding and 5-HT_1B_ binding are highly correlated in most cortical regions, this was not observed for subcortical regions (including putamen), which are lacking 5-HT_1B_ heteroreceptors [46]. In addition, in contrast to 5-HTT, the 5-HT_1B_ receptor is more dynamic: it changes following cognitive behavior therapy, tryptophan depletion or acute SSRI administration for example [76]. Although the 5-HT_1B_ receptor has been associated with social behaviors, these primarily involve states such as anxiety, reward or dependence-driven behaviors [76], rather than a stable trait such as social cognition. While no human studies have been published examining social cognition in relation to the 5-HT_1B_ receptor, autistic mouse model studies demonstrate that administration of a 5-HT_1B_ receptor antagonist can rescue sociability deficits [77]. However, it is questionable whether these social deficits observed in mice accurately reflect human social cognition abilities. Therefore, with no previously reported link to 5-HT_1B_ in human behavior, the dynamic nature of it and our null-finding, we pose that the 5-HT_1B_ receptor is probably not involved in social cognition.

### Limitations

Several limitations warrant consideration. First, the inclusion of 14 controls from the original study violates assumptions for frequentist statistical analysis, raising concerns about the validity of the combined results. However, in the separate replication sample we obtained a correlation between MASC and 5-HTT binding in putamen, although this did not survive multiple comparison correction. The subsequent implementation of Bayes Replication Factors supported a replication of the correlation between MASC and 5-HTT binding in putamen, in contrast to the other brain regions or cognitive tests.

Second, combining datasets from two distinct PET systems can pose several challenges due to variations in system-specific characteristics, such as scanner sensitivity, spatial resolution and reconstruction algorithms. These differences can lead to inconsistencies in the data, affecting the accuracy and reliability of the pooled results. We tried to overcome these PET system differences by a separate analysis and standardization of the datasets. Also, the Bayesian approach is resilient to differences in PET systems, as it utilizes correlation coefficients from each study independently. This ensures that potential variations stemming from PET system disparities do not unduly influence the assessment of the correlation’s reproducibility.

Finally, limited statistical power is an inherent challenge in PET research. The emphasis on the inherent challenges in this research is crucial. The complexity of studying the brain and behaviour, compounded by the inherent limitations in the reliability of PET measures and cognitive testing, underscores the difficulty of drawing definitive conclusions. The potential for false positives (false discovery rate) is commonly estimated as 5% in frequentist statistics. With the examination of 17 brain regions and 5 cognitive tests, the likelihood of obtaining 4–5 significant results is anticipated, as observed in our study. Employing the Bayesian analysis, we were able to identify some possible false positive findings from our original study.

## Conclusions

Our hypothesis, asserting a connection between social cognition and 5-HTT binding, finds partial confirmation. Replication in a sample of healthy controls substantiates our previous findings, with the strongest evidence emerging for putaminal 5-HTT involvement in social cognition within the general population. However, the role of other brain regions remains less certain and warrants further investigation. In contrast, our exploratory analysis revealed no evidence for of 5-HT_1B_ receptor involvement in social cognition. In addition, we found no convincing evidence for 5-HTT or for 5-HT_1B_ involvement in central coherence.

The divergent findings between 5-HTT and 5-HT_1B_ receptor binding are particularly noteworthy and highlight the complexity of serotonergic modulation of social cognition. The observation that a global marker of serotonergic function (5-HTT) correlates with social cognitive performance while a specific receptor subtype (5-HT_1B_) does not suggests several possible mechanisms. First, putaminal involvement in social cognition may be mediated through broad serotonergic tone rather than through specific receptor-mediated pathways. This could indicate that social cognitive performance is more sensitive to overall serotonin availability and reuptake efficiency than to the activation of particular receptor subtypes.

Alternatively, 5-HTT binding may reflect upstream regulatory processes or compensatory mechanisms that influence multiple downstream serotonergic pathways simultaneously, effects that would not be captured by measuring individual receptor subtypes. The global nature of 5-HTT function—regulating synaptic serotonin availability across multiple receptor systems—may be more relevant for complex cognitive processes like social cognition that likely depend on coordinated activity across various serotonergic circuits.

In conclusion, these findings suggest that global serotonergic function, as indexed by 5-HTT binding, may be more relevant than specific receptor mechanisms for social cognitive processes, providing partial support for serotonergic involvement in social cognition even within healthy populations.

## Data Availability

The datasets generated and/or analysed during the current study are available in the Open Science Framework repository, https://osf.io/z6mbn/?view_only=d4028579be2143d3b077f408f65e78ce.

## Abbreviations

5-HT: Serotonin
5-HTT: Serotonin transporter
5-HT_1B_: Serotonin receptor 1B
PET: Positron emission tomography
BP_ND_: Binding potential
BFR: Bayes replication factors
ToM: Theory of mind
R-DoC: Research domain criteria
[^11^C]MADAM: [¹¹C]N, N-Dimethyl-2-(2-amino-4-methylphenylthio)benzylamine (carbon 11)
[¹¹C]AZ10419369: [¹¹C]2-(1,4-Biazepan-1-yl)-N-(4-methoxybenzyl)-N-(1,3-thiazol-4-yl)acetamide
MASC: Movie for the assessment of social cognitio
RMET: Reading the mind in the eyes test
EFT: Embedded figures test
FPT: Fragmented pictures test
GM: Gray matter
PUT: Putamen
BS: Brain stem
FC: Frontal cortex
ACC: Anterior cingulate cortex
INS: Insula
AMG: Amygdala
PCC: Posterior cingulate cortex
NAC: Nucleus accumbens
